# Lack of Effect of the *Salmonella* Deubiquitinase SseL on the NF-κB Pathway

**DOI:** 10.1371/journal.pone.0053064

**Published:** 2013-01-08

**Authors:** Francisco S. Mesquita, David W. Holden, Nathalie Rolhion

**Affiliations:** Section of Microbiology, MRC Centre for Molecular Bacteriology and Infection, Imperial College London, London, United Kingdom; Universite de la Mediterranee, France

## Abstract

Intracellular replication of *Salmonella enterica* requires effector proteins translocated across the *Salmonella*-containing vacuolar membrane by *Salmonella* pathogenicity island-2 (SPI-2) encoded type III secretion system (T3SS). The SPI-2 T3SS effector SseL is a deubiquitinase that contributes to virulence in mice. Previous work has produced conflicting evidence as to the involvement of SseL in interference with the NF-κB pathway. To attempt to clarify these discrepancies, we compared mRNA levels in mouse primary bone marrow-derived macrophages infected with wild-type or *sseL* mutant strains using a genome-wide microarray. There was no detectable effect of loss of SseL on mRNA levels corresponding to any known NF-κB-regulated gene. In addition, there was no effect of SseL on (i) the activation or levels of both the canonical inhibitor of the NF-κB pathway (IκBα and phospho-IκBα), and the non-canonical NF-κB precursor p100/p52, (ii) the translocation of the NF-κB transcription factor p65 to the nucleus of infected macrophages and (iii) pro-inflammatory cytokines secretion. Furthermore, ectopic expression of SseL did not affect NF-κB activation in reporter cell lines. These results fail to support a role for SseL in the down-regulation of the host immune response and in particular the NF-κB pathway.

## Introduction

The nuclear factor kappa-light-chain-enhancer of activated B cells (NF-κB) pathway comprises a family of transcription factors, the NF-κB/Rel proteins, that regulate expression of genes involved in different biological processes such as cell proliferation, immune responses, inflammation and cell death. In resting conditions, these transcription factors remain in the cytosol sequestered by inhibitors, the IκB proteins [1], [2], [3]. Following cellular receptor stimulation, the IKK kinase complex (IKKα, IKKβ, NEMO) is activated through K63-linked polyubiquitination and phosphorylates NF-κB inhibitors [2], [3]. This results in their K48-linked ubiquitination by the SCF^β-TrCP^ E3 ligase complex and consequently proteasomal degradation/processing [2], [3]. The NF-κB transcription factors are then released in the cytosol and translocated to the nucleus to regulate the expression of genes involved in pro-inflammatory signalling. Deubiquitinases (DUBs) can negatively regulate NF-κB signalling through cleavage of K63-linked chains, which form scaffolds for the activation of the IKK kinase complexes [2], [3], [4]. The NF-κB pathway is classified as either classical (canonical) or alternative (non-canonical) on the basis of the IKK subunits that are activated by upstream kinases and which lead to the activation of different NF-κB transcription factors [5], [6].

Many bacterial pathogens, including *Salmonella enterica*, have acquired sophisticated mechanisms to interfere with the NF-κB signalling pathway [7], [8]. *Salmonella* has two type three secretion systems (T3SS), encoded within the *Salmonella* pathogenicity islands (SPIs) 1 and 2 that deliver virulence effector proteins into the host cell. In the case of the SPI-1 T3SS, these mediate bacterial invasion into host cells [9], while the SPI-2 T3SS translocates effectors across the vacuolar membrane of intracellular bacteria to promote replication [10], [11]. SopE, SopE2 and SopB constitute a subset of SPI-1 effectors that are important for invasion and promote intestinal inflammation through the activation of the NF-κB and MAPK pathways independently of immune receptors [12]. AvrA has been reported to inhibit NF-κB activity and pro-inflammatory cytokine secretion [13], [14], [15], but does not seem to interact with or affect the activity of known proteins involved in the NF-κB pathway [16]. The SPI-2 T3SS effector SspH1 binds to the kinase PKN1 [17], which in turn regulates NF-κB and JNK signalling [18], [19]. Constitutively active PKN1 and SspH1 were shown to negatively regulate NF-κB signalling when expressed ectopically in epithelial cells [17].

Our group showed that the *Salmonella* Typhimurium SPI-2 T3SS effector SseL is a DUB with a preference for K63-linked chains and that it contributes to macrophage cell death, but no difference in degradation of IκBα or production of the pro-inflammatory cytokine TNF-α was detected in macrophages infected with either wild-type (wt) or *sseL* mutant strain of *S.* Typhimurium [20]. A subsequent study proposed that SseL deubiquitinates IκBα, thereby preventing its degradation and reducing NF-κB signalling [21]. SseL was also suggested to reduce innate immune responses *in vivo* [21]. Due to the discrepancies between this work and our previous results, we carried out further experiments on the potential influence of SseL on the NF-κB pathway. Our results fail to provide evidence that SseL targets the NF-κB pathway.

## Results

### Microarray analysis of macrophage mRNAs

**Figure 1:**
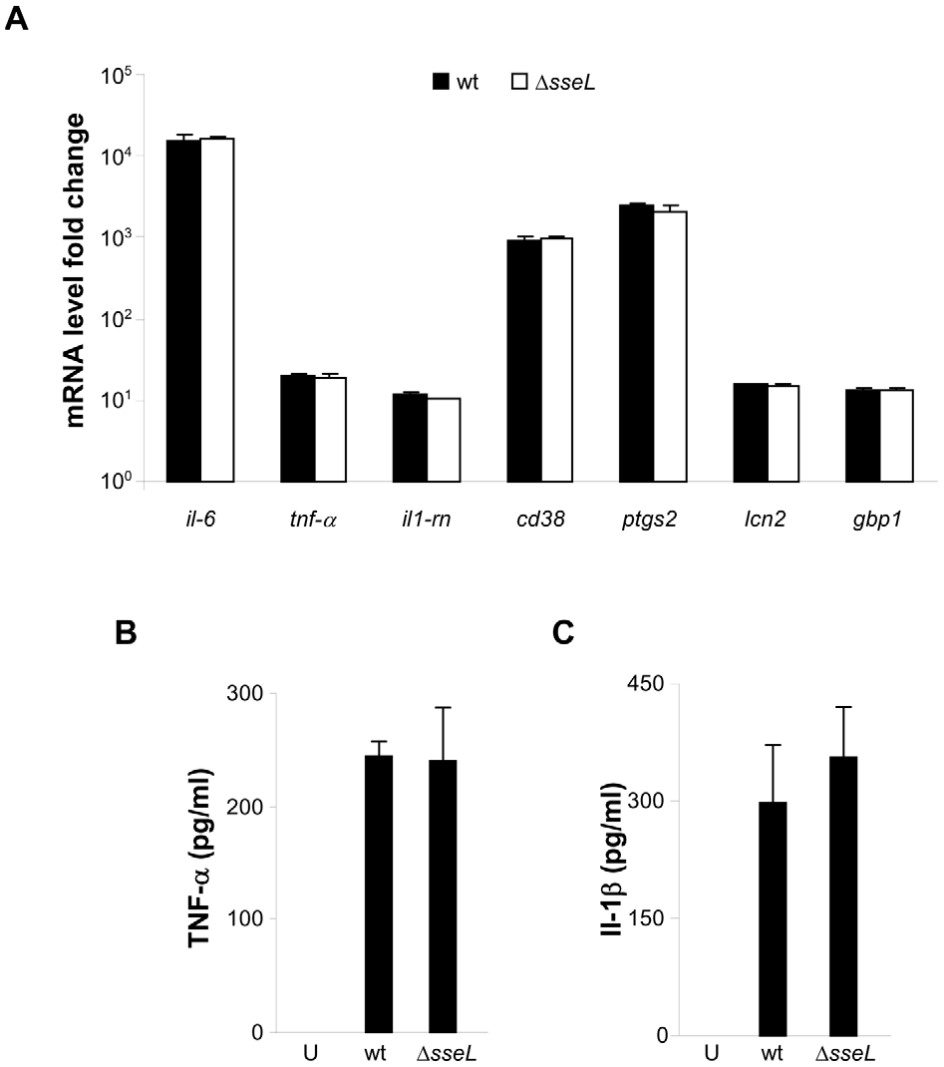
SseL does not influence NF-κB regulated gene expression and secretion of TNF-α and Il-1β in infected primary macrophages. (A) BMMs were infected wt (black bars) or Δ*sseL* (white bars) strains and mRNA levels of *il-6*, *tnf*-α, *il1-rn*, *cd38*, *ptgs2*, *lcn2* and *gbp1* gene were analysed by qRT-PCR after reverse transcription of RNA extracted from cells. The transcript levels were normalized to the levels of *rsp9*, which were constant under all conditions used, and expressed relative to those of uninfected macrophages. Results are expressed as mean ± SEM of 3 independent experiments. Levels of secreted TNF-α (B) and Il-1β (C) were quantified by ELISA in supernatants of uninfected (U) BMMs or BMMs infected with indicated strains of *S.* Typhimurium at 10 h or 24 h post-uptake respectively. Results presented are the means ± SEM of triplicates of one experiment. These results are representative of three independent experiments.

To broaden the analysis of a possible role of SseL in immune modulation, we first analysed variations in mRNA levels from uninfected bone marrow-derived macrophages (BMM) and BMM infected with either wt or Δ*sseL* strains at 10 h post-bacterial uptake using a genome-wide DNA microarray representing 28853 genes. BMM were chosen as they provide a more physiological environment for *S.* Typhimurium than macrophage-like cell lines. Immunofluorescence microscopy confirmed that translocation of SseL into the BMM cytosol occurred by this time-point ([20], [22], unpublished work) but overall growth of wt and Δ*sseL* mutant bacteria was indistinguishable ([23], unpublished work). Infection of BMM with wt or Δ*sseL* bacteria led to very dramatic changes in mRNA levels in comparison to uninfected cells (Table 1). However, there was no detectable difference in any gene, including NF-κB-regulated genes, between macrophages infected with wt or Δ*sseL* mutant strains (P>0.05) (Table 1). The NF-κB-regulated genes showing the highest fold change in expression in infected macrophages are shown in Table 1. To confirm the data obtained by microarray analysis, quantitative real-time PCR (qRT-PCR) was conducted on selected NF-κB-regulated genes. The levels of *il-6*, *tnf*-α, *il1-rn*, *cd38*, *ptgs2*, *lcn2* and *gbp1* mRNA were equivalent in macrophages infected with either strain (Fig. 1A), and these data were in agreement with the microarray data. Moreover, infection with Δ*sseL* mutant bacteria did not cause macrophages to secrete more TNF-α (at 10 h post-uptake) and Il-1β (at 24 h post-uptake) when compared to macrophages infected with the wt strain (Fig. 1B and C). Together, these results fail to provide evidence that SseL modulates the mRNA levels of NF-κB-regulated genes or pro-inflammatory cytokines secretion.

**Table 1:** NF-κB-regulated mRNAs altered by *Salmonella* infection.

Gene symbol	Fold change induced by wt	Fold change induced by Δ*sseL*	Protein or RNA
**Cytokines/chemokines**			
*il-6*	+219.32	+202.21	interleukin-6
*il-1α*	+175.35	+168.39	interleukin-1 alpha
*cxcl3*	+84.94	+83.32	chemokine (C-X-C) ligand 3
*il-12β*	+43.89	+38.56	Interleukin-12 beta
*il-1β*	+26.67	+26.60	Interleukin-1 beta
*cxcl1*	+17.99	+17.75	chemokine (C-X-C) ligand 1
*tnf-α*	+16.20	+15.91	tumor necrosis factor alpha
*saa3*	+14.87	+14.96	serum amyloid A protein
*il1-rn*	+14.73	+15.13	Interleukin-1 receptor antagonist
*ccl5*	+14.36	+14.33	chemokine (C-C) ligand 5
*ccl4*	+13.32	+12.24	chemokine (C-C) ligand 4
*il-12α*	+10.62	+10.28	Interleukin-12 alpha
**Immunoreceptors**			
*cd38*	+57.97	+59.42	cluster of differentiation 38
*cd40*	+24.33	+24.25	cluster of differentiation 40
*trem1*	+13.97	+12.99	triggering receptor expressed on myeloid cells 1
**Stress response**			
*ptgs2*	+107.36	+107.1	prostaglandin-endoperoxide synthase 2
*inos2*	+24.06	+25.55	inducible nitric oxide synthase 2
**Antigen presentation**			
*cfb*	+13.37	+13.42	complement factor B
**Cell adhesion**			
*tnc*	+19.56	+19.89	tenascin
**Cell surface receptor**			
*cd69*	+17.03	+15.87	cluster of differentiation 69
**Regulator of apoptosis**			
*bcl2a1b*	+11.25	+11.06	B-cell leukemia/lymphoma 2 related protein A1b
**Enzyme**			
*ptges*	+17.89	+18.62	prostaglandin E synthase
**Miscellaneous**			
*miR155*	+24.93	+24.66	microRNA 155
*edn1*	+19.88	+18.20	endothelin 1
*lcn2*	+19.41	+19.30	lipocalin 2
*gcnt1*	−18.12	−18.23	glucosaminyl transferase 1
*gpb1*	+11.62	+11.73	guanylate binding protein 1

Fold changes were calculated for each strain relative to the uninfected control and values represent the mean of an experiment done in triplicate.

### Lack of effect of SseL on IκBα phosphorylation and degradation in infected macrophages

**Figure 2:**
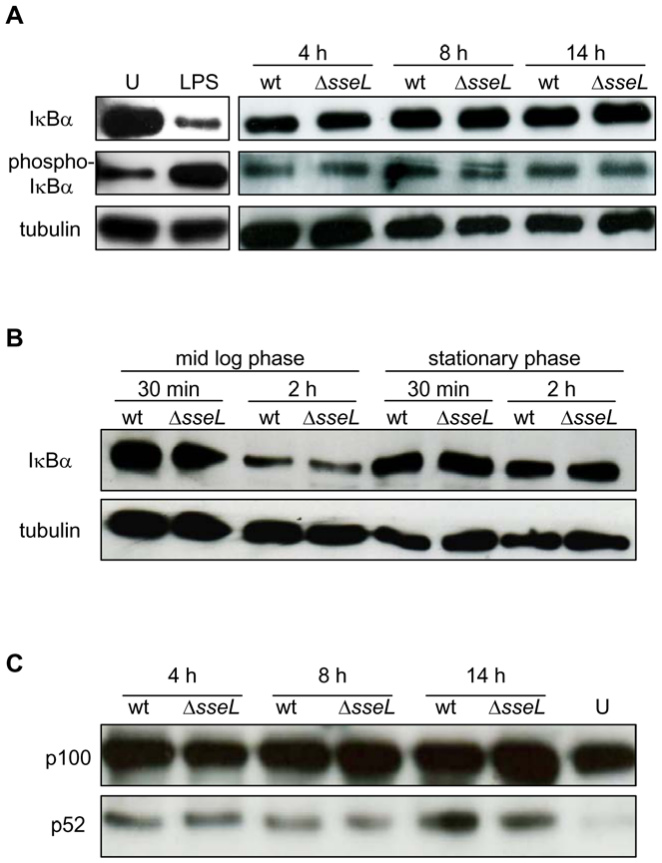
SseL does not influence degradation and processing of IκBα and p100 in infected primary macrophages. Macrophages were uninfected (U), exposed to LPS (1 µg/ml) for 2 h or infected with indicated strains of *S.* Typhimurium. (A) Cells were harvested and lysed at the indicated time-points after bacterial uptake and the levels of IκBα and phospho-IκBα were analysed by immunoblot using anti-IκBα and anti-phospho-IκBα antibodies. The same blots were probed for β-tubulin as a loading control. Immunoblots are representative of three independent experiments. (B) Macrophages were infected with *S*. Typhimurium strains (wt or Δ*sseL* mutant) grown to mid-log or stationary phase. Cell lysates were collected at different time-points after bacterial uptake and analysed for IκBα levels by immunoblot using anti-IκBα antibody. The membrane was reprobed with an anti-β-tubulin antibody, as a loading control. Immunoblots are representative of three independent experiments. (C) Macrophages were infected as in (A) and cell lysates were collected at different time-points after bacterial uptake and analysed by immunoblot using anti-p100/p52 antibody. U lane represents uninfected cells. Results are representative of three independent experiments.

In an attempt to address the discrepancies between our results [20] and the study of Le Negrate *et al.* [21], we re-analysed the effect of SseL on different steps of the NF-κB-pathway activity. Analysis of the levels of IκBα is commonly used as a measure of the activation of the canonical NF-κB pathway [13], [15], [16], [24]. Our group previously analysed the levels of IκBα and phospho-IκBα in J774 macrophages infected with wt or *sseL* mutant bacteria and did not observe any difference in protein activation or protein levels of IκBα up to 22 h after bacterial uptake [20]. We extended the analysis of the potential effect of SseL on degradation of IκBα in primary BMM at different time-points after bacterial uptake. Lysates from murine BMM infected with wt or Δ*sseL* mutant strains were obtained at 4 h, 8 h and 14 h after bacterial uptake and analysed for the levels of IκBα and phospho- IκBα by immunoblot (Fig. 2A). Cells stimulated with LPS for 2 h had markedly decreased levels of IκBα and increased phosphorylation of IκBα when compared to uninfected cells, showing that these cells responded to an agonist of the NF-κB pathway (Fig. 2A). However, the levels of both IκBα and phospho-IκBα in BMM infected with wt or Δ*sseL* mutant strain were indistinguishable at the three time-points analysed (Fig. 2A).

Le Negrate *et al* (2008) used mid-log cultures of *S.* Typhimurium to infect BMM for 30 min prior to analysis [21]. We attempted to reproduce these assays in BMM using either bacteria grown until mid-log phase (when bacteria express SPI-1 genes [25]) or stationary phase (when bacteria repress SPI-1 genes and express SPI-2 genes [25]) and harvesting macrophages at both 30 min and 2 h after bacterial uptake. In agreement with our results obtained with macrophages harvested at later time points post-bacterial uptake (Fig. 2A), there was no detectable difference between the levels of IκBα in macrophages infected with wt or Δ*sseL* mutant bacteria at earlier time-points, using mid-log or stationary phase bacterial cultures (Fig. 2B). Interestingly, when macrophages were infected for 2 h with bacteria grown until mid-log phase, the levels of IκBα were reduced when compared to those in macrophages infected with bacteria grown to stationary phase (Fig. 2B). This indicates stronger activation of the NF-κB pathway by bacteria in mid-log phase of growth and is in agreement with reports showing that a subset of SPI-1 T3SS effectors and bacterial flagella activate the NF-κB pathway [12], [26]. Together these results do not provide any evidence for a role of SseL in the phosphorylation and degradation of the inhibitor of the canonical NF-κB pathway, IκBα.

### Processing of p100 is not affected by SseL

Both the classical and alternative signalling pathways can lead to the transcription of NF-κB-regulated genes. The alternative pathway involves ubiquitin-dependent processing of the IκB precursor protein p100 into the NF-κB transcription factor p52 and responds to stimuli from a small subset of TNF family members such as the LTαβ, B cell activating factor (BAFF), CD40L and TWEAK (TNF-related weak inducer of apoptosis) and also to LPS [2], [27], [28]. To determine the potential effect of SseL on signalling transduced through the alternative NF-κB pathway, the processing of p100 into p52 in BMM infected with wt or Δ*sseL* mutant bacteria was analysed. p100 was processed into p52 in samples from cells infected by wt or Δ*sseL* mutant bacteria at each of the three time-points analysed, but not in uninfected cells (Fig. 2C). This shows that *Salmonella* activates the alternative NF-κB pathway, but fails to provide evidence that SseL influences the processing of p100.

### Nuclear translocation of p65 in infected macrophages

**Figure 3:**
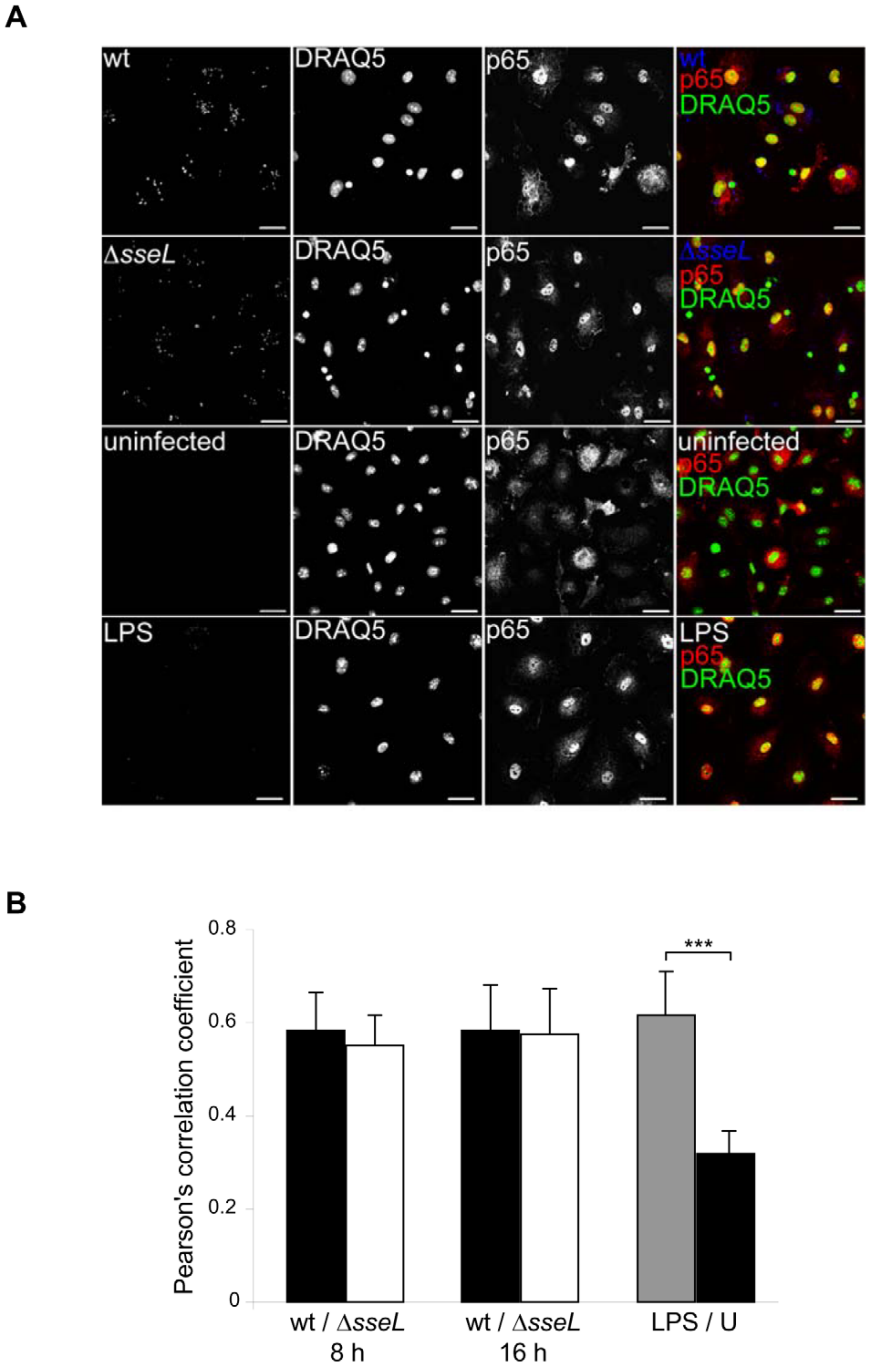
SseL does not influence localization of p65 in infected primary macrophages. Macrophages were infected with the indicated strains of *S*.Typhimurium. Cells were fixed at 8 h and 16 h after bacterial uptake, immunolabelled using anti-*Salmonella* and anti-p65 antibodies and stained with DNA dye DRAQ5. Samples were analysed by confocal microscopy and false coloured using Zeiss LSM image software (scale bars - 20 µm). (A) Representative examples from the 8 h time-points and the controls (uninfected macrophages and macrophages exposed to LPS (1 µg/ml) for 2 h). (B) Quantification of p65 nuclear translocation using Pearson's correlation coefficient between p65 and DRAQ5 in individual cells. At least 50 cells were quantified in each sample at each time point. Values represent the mean ± SD of a representative experiment. P-values were obtained using two-tailed unpaired Student's *t*-test (***P<0.001).

Some T3SS effectors inhibit NF-κB activation by blocking nuclear translocation of NF-κB subunits [29]. To determine the potential effect of SseL on nuclear translocation of the canonical NF-κB transcription factor p65, confocal microscopy was used to quantify the translocation of p65 into the nucleus of murine BMM infected with the wt or Δ*sseL* mutant strains at 8 h and 16 h post-uptake. Cells were immunolabelled with anti-*Salmonella* and anti-p65 antibodies and stained with the DNA dye, DRAQ5 (Fig. 3A). LPS-stimulated macrophages and uninfected cells were used as controls (Fig. 3A). Three-dimensional image projections were acquired and levels of translocation of p65 to the nucleus were measured by quantifying co-localization between p65 and DRAQ5 in individual cells. There was, as expected, significantly more colocalization between p65 and DRAQ5 in LPS-treated macrophages compared to uninfected cells at 2 h post-challenge (Fig. 3B). In contrast, the colocalization between p65 and DRAQ5 in BMM infected with wt or Δ*sseL* mutant strains was equivalent at both time-points analysed (Fig. 3B). Similar results were obtained in infection assays using the J774 macrophage-like cell line (unpublished work). Therefore, we conclude that SseL does not influence the translocation of p65 to the nucleus in infected macrophages.

### Activation of an NF-κB regulated promoter is not affected by SseL

**Figure 4:**
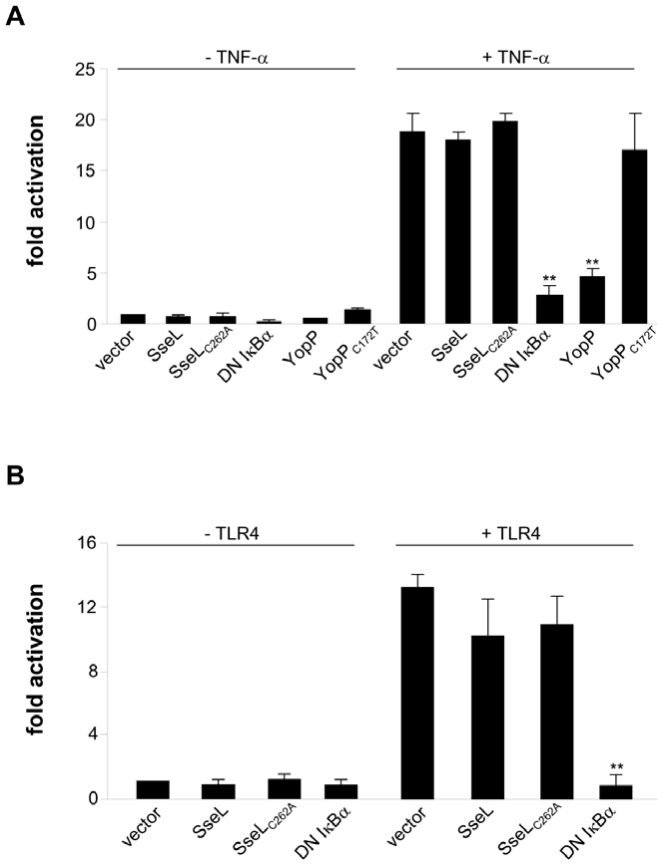
SseL does not influence NF-κB activation. (A) HEK293 cells were co-transfected with NF-κB reporter plasmids and vector alone or vectors expressing myc-SseL, myc-SseL_C262A_, myc-YopP, myc-YopP_C172T_ (inactive YopP) or dominant negative IκBα (DN IκBα) for 16 h. The NF-κB pathway was activated with TNF-α and luciferase activity was measured after 8 h and expressed as fold activation in relation to unstimulated cells transfected with vector alone. Values are expressed as mean ± SEM of 3 independent experiments. P-values were obtained using two-tailed unpaired Student's t-test and are relative to the mean value of the corresponding cells transfected with vector alone (**P<0.01). (B) HEK293 cells were co-transfected with NF-κB reporter plasmids, vector alone or vectors expressing myc-SseL, myc-SseL_C262A_ or dominant negative IκBα (DN IκBα) and a vector encoding a constitutively active LPS receptor TLR4 (to activate the NF-κB pathway). Reporter activity was measured 24 h after transfection and expressed as fold activation in relation to unstimulated cells transfected with vector alone. Values are expressed as mean ± SEM of 3 independent experiments. P-values were obtained using two-tailed unpaired Student's *t*-test and are relative to the mean value of the corresponding cells transfected with vector alone (**P<0.01).

SseL was shown to modulate NF-κB activation after its ectopic expression in NF-κB reporter cell lines [21]. We established similar assays to try to confirm this effect in cells expressing the luciferase reporter gene under the control of NF-κB binding sequences. HEK 293 cells were co-transfected with vectors expressing myc-SseL or myc-SseL_C262A_ (SseL carrying an amino acid substitution at its catalytic cysteine that abrogates its DUB activity [20]) or myc vector alone (empty vector), along with vectors expressing the luciferase reporter gene under the control of NF-κB promoters, and vectors constitutively expressing the Renilla luciferase gene, for normalization of transfection efficiencies. The NF-κB pathway was stimulated with TNF-α (8 h, 10 ng/ml) or by co-transfection with a vector expressing constitutively active LPS receptor TLR4 (CD16::TLR4 [30]). Transfection with vectors encoding a dominant negative form of IκBα (DN IκBα) or myc-YopP (an *Yersinia* effector that negatively regulates the NF-κB pathway [31], [32], [33]) or its catalytically inactive form (myc-YopP_C172T_) were used as controls (Fig. 4). After stimulation, cells were lysed and bioluminescence was measured to determine the fold-difference of NF-κB activation in relation to non-activated HEK 293 cells transfected with myc vector alone (Fig. 4). We verified that we used equivalent numbers of cells expressing similar levels of effectors (myc-SseL, myc-SseL_C262A_, myc-YopP or myc-YopP_C172T_) by immunoblot using anti-tubulin and anti-myc and we checked the DUB activity of SseL by immunoblot using anti-mono-ubiquitinated and poly-ubiquitinated proteins antibodies (unpublished work). Cells transfected with the empty vector displayed an approximately 20-fold increase in activation following stimulation with TNF-α (Fig. 4A) and an approximately 15-fold increase with constitutively active TLR4 (Fig. 4B), when compared to resting cells. Ectopic expression of a dominant negative form of IκBα (Fig. 4A and 4B) or YopP (Fig. 4A) resulted in a strong inhibition of NF-κB activation. As expected, transfection of YopP_C172T_ did not inhibit NF-κB activation (Fig. 4A). Cells expressing SseL or SseL_C262A_ displayed no reduction in NF-κB activation after stimulation with LPS or when expressing constitutively active TLR4, when compared to control cells (Fig. 4). Together, these results fail to show that SseL inhibits the NF-κB pathway.

## Discussion

This study failed to confirm the findings of Le Negrate *et al.* who reported that SseL inhibits the NF-κB pathway [21]. Firstly, an unbiased approach using a DNA microarray to assess changes in mRNAs levels in response to infection by wt or *sseL* mutant bacteria did not reveal any obvious differences for genes regulated by NF-κB transcription factors. Secondly, the release of pro-inflammatory cytokines was also unaffected by deletion of SseL. Thirdly, infection of BMM did not provide evidence to support an effect of SseL on key steps of NF-κB signalling: phosphorylation and degradation of IκBα, processing of the precursor of the alternative NF-κB pathway p100 or the nuclear translocation of p65. Finally, production of SseL in reporter cells did not affect NF-κB pathway signalling.

In each of these assays, the NF-κB pathway was shown to be activated by different stimuli. Therefore our negative results cannot be explained by a general lack of responsiveness of the host cells. Furthermore, we found that production of the *Yersinia* effector protein YopP after transfection was efficient in reducing NF-κB signalling, as previously described [32], [33], showing that the assay was sufficiently sensitive to detect effects on NF-κB signalling.

One explanation for the discrepancies between the results observed in our reporter cell assays and those carried by Le Negrate *et al.* could be the conditions used to ectopically express SseL [21]. In their assays, Le Negrate *et al.* waited until 72 h after transfection of HEK293T cells to stimulate them with TNF-α or Il-1β and then waited 6 h before assaying transcriptional responses. We incubated the same cell type with TNF-α for 8 h at 16 h post-transfection, prior to cell lysis. Le Negrate *et al.* also used greater amounts of DNA to transfect fewer cells. Therefore it is possible that these differences could account for our failure to detect an effect of SseL. However it is also possible that increased and extended expression of SseL might cause non-specific effects leading to deubiquitination of IκBα and reduced NF-κB signalling. Non-specific targeting of the NF-κB pathway has already been reported for the ectopic overexpression of the *Salmonella* effector AvrA in mammalian cells [16].

It is less clear how discrepancies between the results from macrophage infection assays can be explained. It remains possible that the *sseL* mutant strain used by Le Negrate *et al.* could carry additional adventitious mutations that may have influenced NF-κB activation or that insertion of the kanamycin cassette in the *sseL* gene could have a polar effect on upstream and/or downstream genes. Alternatively, differences in the infection rates between macrophages infected with wt or Δ*sseL* mutant bacteria could account for variations in NF-κB stimulation. Another possible explanation relates to the growth phase of bacteria that were used to infect macrophages. Le Negrate *et al.* used bacteria that were likely to be in mid-log phase. Under these conditions, the SPI-1 T3SS is expressed but the SPI-2 T3SS is not activated [25]. In our experiments, we used bacteria grown in mid-log and also stationary phase and we were not able to detect any effect of the lack of SseL in any conditions tested. Le Negrate *et al.* reported the deubiquitination of IκBα by SseL in BMM infected with bacteria grown until mid-log phase 30 min after bacterial uptake [21]. This is very surprising in view of work showing that (i) SseL is a SPI-2 T3SS-specific effector [20], [22], [34]; (ii) SPI-2 T3SS gene expression is induced after 90 min post-uptake of bacteria in macrophages [35] and (iii) translocated SseL was only detectable by immunofluorescence microscopy 6 h after bacterial entry [20].

Two independent competitive index tests demonstrated that SseL contributes to systemic growth of bacteria in wt mice [20], [22]. Using a streptomycin-treated mouse model, Le Negrate *et. al*. did not detect a growth defect of Δ*sseL* mutant bacteria but reported that mice infected with this strain had an increased production of inflammatory cytokines and greater tissue inflammation in the gut, spleen and liver [21]. Streptomycin-treated mice provide a model for studying gastrointestinal disease, but antibiotic treatment is not necessary for the induction of systemic infection in BALB/c mice. It is possible that residual antibiotic interfered with bacterial growth in the spleens and livers, altering the disease progression and thereby preventing the determination of an effect of SseL on bacterial growth.

Recent work from our group showed that the deubiquitinase activity of SseL prevented accumulation of aggresome-like induced structures (ALIS) and other ubiquitinated aggregates in infected epithelial cells and macrophages, thereby reducing the host autophagic response [23]. In addition, SseL was shown to bind oxysterol-binding protein (OSBP) [36], [37] and to alter lipid metabolism in infected cells [38]. However, these studies do not provide an obvious link to the possible role of SseL on NF-κB activation.

In summary, although we used different approaches to test the involvement of SseL in modulation of host immune responses, none of our results support the hypothesis that it is involved in inhibition of host inflammatory responses.

## Materials and Methods

### Bacterial strains, plasmids and growth conditions

*Salmonella enterica* serovar Typhimurium wild-type 12023 (NTCC, Colindale, UK) and a Δ*sseL* derivative [20] and *Escherichia coli* (Invitrogen) were grown in Luria Bertani (LB) medium, at 37°C with aeration. When appropriate, bacterial cultures were supplemented with antibiotics: 50 µg/ml for kanamycin (Km) and ampicillin (Amp). All plasmids used in this work are listed in Table S1.

### Antibodies and Dyes

For immunofluorescence microscopy, the rabbit anti-p65 (Santa Cruz) was used at 1∶200; the CSA-1 goat anti-*Salmonella* (Kirkegaard and Perry Laboratories) at 1∶400; the DNA dye DRAQ5 (Alexis) at 1∶200. Secondary antibodies were obtained from Invitrogen: Alexa 488-, Alexa 555-conjugated donkey anti-goat, or anti-rabbit, were used for immunofluorescence at a dilution of 1∶400. For immunoblotting, antibodies were used at the following dilutions: the rabbit anti-IκBα (Cell Signalling) at 1∶1000, the rabbit anti-phospho-IκBα (Cell Signalling) at 1∶1000; the rabbit anti-p100/p52 (NIMR – MRC) at 1∶1000; the E7 mouse anti-β-tubulin (Developmental Studies Hybridoma Bank) at 1∶1000; the FK2 mouse anti-mono- and poly-ubiquitinated proteins (ENZO) at 1∶1000 and anti-mouse and anti-rabbit (IgG) horseradish peroxidase secondary antibodies (GE Healthcare) at 1∶10000.

### Cell Culture and Transfection

Human Embryonic Kidney 293 cells (HEK293) were kindly provided by Felix Randow from the Laboratory of Molecular Biology Cambridge University, UK. Cells were grown in Dulbecco's modified Eagle medium (DMEM, PAA laboratories) supplemented with 10% heat inactivated foetal calf serum (FCS, PAA laboratories) at 37°C in 5*%* CO_2_.

BMM were obtained from BALB/c mice, extracted from tibia and femur [39] and grown as described [10].

### Transfection and reporter assays

HEK293 cells were transfected using Lipofectamine 2000 transfection reagent (Invitrogen) in accordance with the manufacturer's instructions. Cells were seeded at density of 5×10^4^ cells per well in a 24-well plate 16 h prior to transfection. For TNF-α activation, cells were transfected for 16 h with luciferase reporter plasmid (50 ng), 30 ng of pTK-Renilla luciferase, and 100 ng of expression vectors (myc-SseL; myc-SseL_C262A_, myc-YopP, myc-YopP_C172T_ or myc vector alone) or 100 ng of pEAK12-dominant negative IκB. Cells were then incubated with 10 ng/ml of TNF-α for 8 h and harvested in 100 µl of passive lysis buffer (Promega). In the case of TLR4 stimulation, cells were transfected for 24 h as described above and also with 100 ng of pucEDV-TLR4::CD16 vectors (which express a constitutively active LPS receptor). Cells were then harvested in 100 µl of passive lysis buffer. Luciferase activity was measured using Dual Luciferase reporter assay system (Promega) and a TD20/20 Luminometer (Turner Designs) and normalised according to Renilla luciferase intensity. The data presented are from at least three independent experiments.

### Bacterial infections

All macrophage infections were done as previously described [40] except for infections using mid-log cultures of *S.* Typhimurium which were done with bacteria incubated for 16 h at 37°C with shaking, diluted 1∶33 in fresh LB broth and incubated in the same conditions for 3.5 h.

### Immunofluorescence microscopy

For immunofluorescence microscopy, uninfected cells and cells subjected to LPS (1 µg/ml) stimulation for 2 h were used as controls. All samples were fixed in 4% paraformaldehyde and permeabilized in 0.2% Triton X-100 for 5 min. All antibodies were diluted to the appropriate concentration in PBS containing 10% horse serum. The coverslips were washed twice in phosphate buffer saline (PBS), incubated with primary antibodies for 1 h, washed 3 times in PBS, incubated with secondary antibodies for 30 min and stained with the nucleic acid dye, DRAQ5 for 20 min. Coverslips were washed and mounted on to glass slides using Mowiol mounting medium. Cells were observed with a confocal laser scanning microscope (Zeiss Axiovert LSM510).

### Quantification of nuclear translocation of p65 in infected primary macrophages

Confocal three-dimensional projections were acquired for each sample using a slice increment of 0.4 µm. The co-localization between p65 and DRAQ5 in individual cells was measured using Volocity image software analysis for at least 50 cells for each sample and expressed by Pearson's correlation coefficient. This coefficient provides a measure of correlation between two variables (total p65 labelling and the nucleus, stained by DRAQ5), giving a value between +1 and −1. A value of 0 indicates no linear correlation between the variables.

### Immunoblot analysis

For immunoblot analysis of the levels of IκBα, phospho-IκBα and p100/p52, infected BMM (6×10^6^ cells) were washed and harvested in ice cold PBS and centrifuged at 300× *g* for 2 min at different time-points. Uninfected cells and cells subjected to LPS (1 µg/ml) stimulation for 2 h before harvesting were used as controls. Cells were lysed in sample buffer, heated for 5 min at 100°C and proteins in lysates were separated using 10% polyacrylamide gels by SDS-PAGE followed by immunoblot analysis.

### RNA extraction, microarray analysis and qRT-PCR

At 10 h post-uptake, cells were washed and RNA was isolated using TRIzol according to the manufacturer's directions (Invitrogen). Contaminating genomic DNA was removed using DNaseI (Qiagen). Labelled cDNA was synthesized in triplicate from 200 ng of RNA and hybridized to mouse gene 1.0 ST arrays (Affymetrix). These arrays provide whole-transcript coverage, with each of 28853 genes represented on the array by approximately 27 probes spread across the full length of the gene. Data were analysed with Agilent GeneSpring GX software. For qRT-PCR, RNAs (400 ng) were reverse transcribed with Quantiscript Reverse transcriptase (QuantiTect Reverse Transcription kit, Qiagen) for 25 min at 42°C. Quantification of the mRNA levels was done using SensiMix dT kit (Quantace) and specific primers (Table S2) on Rotor-Gene 3000 (Corbett Research).

### Enzyme-Linked Immunosorbent Assay for TNF-α and IL1-β quantification

At 10 h or 24 h post-uptake, supernatants from infected BMM were collected, centrifuged and stored at −80°C. The amount of TNF-α or Il1-β released in the culture supernatant was determined by enzyme-linked immunosorbent assay (ELISA; R&D Systems) and cytokine concentrations were assessed according to the manufacturer's instructions.

## Supporting Information

Table S1 Plasmids used in this work.
(DOCX)

Table S2 Primers used in this work.
(DOCX)
