# Supplementary material for: Lack of Effect of the Salmonella Deubiquitinase SseL on the NF-κB Pathway
Source: PLoS One. 2013 Jan 8;8(1):e53064. doi: 10.1371/journal.pone.0053064 (PMC3540083; doi:10.1371/journal.pone.0053064)
Supplement: Table S1 — Plasmids used in this work. (DOCX) [file pone.0053064.s001.docx]

**Table S1.**

| **Plasmid** | **Description** | **Source or reference** |
| --- | --- | --- |
| pRK5::myc -*sseL* | myc fused to *sseL* | [20] |
| pRK5::myc-*sseL_C262A_* | myc fused to *sseL_C262A_* | [20] |
| M_3_psinrevκB-*luc* | *luc* gene under control of NF-κB consensus promoter | Gift from F. Randow |
| pRLTK | Constitutively active Renilla luciferase | Gift from F. Randow |
| pucEDV-CD16::TLR4 | Constitutively active CD16::TLR4 fusion | [30] |
| pEAK12-DnegIκB | Dominant negative IκB | Gift from F. Randow |
| pGD2::*myc-yopP* | myc fused to YopP in pEF6 | [31] |
| pGD3::*myc-yopP_C172T_* | myc fused to YopP_C172T_ in pEF6 | Gift from G. Cornellis |
