# Supplementary material for: Lack of Effect of the Salmonella Deubiquitinase SseL on the NF-κB Pathway
Source: PLoS One. 2013 Jan 8;8(1):e53064. doi: 10.1371/journal.pone.0053064 (PMC3540083; doi:10.1371/journal.pone.0053064)
Supplement: Table S2 — Primers used in this work. (DOCX) [file pone.0053064.s002.docx]

**Table S2.**

| **Primer** | **Sequence** |
| --- | --- |
| *rsp9 1* | CTGGACGAGGGCAAGATGAAGC |
| *rsp9 2* | TGACGTTGGCGGATGAGCACA |
| *iIl-6 1* | TGGAGTCACAGAAGGAGTGGCTAAG |
| *iIl-6 1* | TCTGACCACAGTGAGGAATGTCCAC |
| *tnf-α 1* | GGCAGGTCTACTTTGGAGTCA |
| *tnf-α 2* | ACATTCGAGGC TCCAGTGAAT |
| *il1-rn 1* | GCAAGCCTTCAGAATCTGGGATAC |
| *il1-rn 2* | CTCAGATCAGTGATGTTAACTTCC |
| *cd38 1* | CATCTACACTCAGATCCTCC |
| *cd38 2* | CCAATTTAACAAGTGGGGCG |
| *ptgs2 1* | TTTGTTGAGTCATTCACCAGACAG |
| *ptgs2 2* | AGGGCTTTCAATTCTGCAGC |
| *lcn2 1* | CCAGTTCGCCATGGTATTTTTC |
| *lcn2 2* | CACACTCACCACCCATTCAGTT |
| *gbp1 1* | ATCACTCCTGATGAGTACCTGG |
| *gbp1 2* | AACTTCCTGATACACAGGCGAG |
